# Diverticular Disease-associated Colitis: What Do We Know? A Review of Literature

**DOI:** 10.7759/cureus.2224

**Published:** 2018-02-24

**Authors:** Fady G. Haddad, Sandy El Bitar, Hassan Al Moussawi, Qing Chang, Liliane Deeb

**Affiliations:** 1 Department of Gastroenterology and Hepatology, Staten Island University Hospital, New York; 2 Department of Medicine, Staten Island University Hospital/northwell Health, New York; 3 Department of Medicine, Staten Island University Hospital, New York; 4 Department of Pathology, Staten Island University Hospital, New York; 5 Department of Gastroenterology, Staten Island University Hospital, New York

**Keywords:** diverticular disease, granulomas, colonic diverticulitis, segmental colitis

## Abstract

Diverticular disease (DD) is a leading cause of hospitalizations in developed countries affecting 30-50% of individuals older than 60 years. Identified as a distinct entity since 1980, diverticular disease-associated colitis (DAC) describes the occurrence of mucosal inflammation in a colon segment affected with DD with relative sparing of the rectum and proximal colon. Its prevalence is suggested around 1.3-3.8%. Pathogenesis is multifactorial with multiple reports noting clinicopathological overlap between DAC and inflammatory bowel disease (IBD) especially in patients with granulomatous colitis. In this setting, caution should be exercised to avoid an inappropriate diagnosis of IBD. Recurrence rates and long-term outcomes of DAC are not well defined and could range from a benign course to an overt IBD. More studies are needed in order to further characterize this entity.

## Introduction and background

Diverticular disease (DD) includes a broad spectrum of manifestations. Diverticular disease-associated colitis (DAC) is a distinct entity that can demonstrate granulomatous reaction. DAC usually affects the left colon and poses a diagnostic and therapeutic dilemma in view of its multiple similarities with inflammatory bowel diseases (IBD). We underwent this review of the literature to further characterize this entity.

## Review

DD is a leading cause of hospitalizations and health care costs in developed countries affecting 30-50% of individuals above 60 years of age. It was considered the third most common cause of gastrointestinal (GI) illness requiring hospitalization and a principal indication for colonic resection according to the 2012 update of the “Burden of gastrointestinal disease in the United States” paper. Recently chronic symptomatic DD has been categorized into chronic recurrent diverticulitis and DAC, also called segmental colitis associated with diverticulosis (SCAD). A third category entitled symptomatic uncomplicated diverticular disease (SUDD) has been described but is not well defined yet [1-3].

Characteristics

DAC has been identified as a distinct entity since 1980 and since then, over 100 cases of DAC have been reported in the literature. It describes the occurrence of mucosal inflammation in a colon segment affected with diverticular disease with relative sparing of the rectum and proximal colon. The occurrence of this entity is not well-defined because of the absence of strict criteria for diagnosis but it is suggested that it has a prevalence of 1.3-3.8%. It usually affects individuals in their sixth or seventh decade, same age of onset of the second peak of IBD. It is suggested that the pathogenesis of DAC is multifactorial. The initial inciting event is the stasis caused by the diverticulum leading to dysbiosis with subsequent immune reaction in certain individuals. In addition, the subserosal inflammation and suppuration in proximity to the diverticulum may lead depending on the patient’s underlying genetic susceptibility to a localized, ulcerative colitis (UC)-like or Crohn’s disease (CD)-like inflammation [1-3].

Clinical and endoscopic findings

DAC presents with non-specific gastrointestinal symptoms including diarrhea being the most common, followed by abdominal and rectal bleeding. Bowel obstruction is also described. The differential diagnosis in adult patients in this particular setting is broad and can include volvulus, intussusception, pseudo-obstruction, ileus among others. Thus radiological, endoscopic, and histopathological techniques are often required to differentiate DAC from other diagnoses. Endoscopic findings reveal hyperemia, granularity, and friability of the colonic mucosa, with diffuse or patched involvement. Three main models of luminal inflammation have been reported in patients with diverticular disease. The first model describes patients with clinical presentation consistent with diverticulitis requiring resection. Coexisting Crohn’s disease was also reported in some individuals in this setting. The second model includes patients with DD who have symptoms due to mucosal inflammation which can be secondary to primary IBD like inflammation limited to areas with diverticulae, nonsteroidal anti-inflammatory drug use, mucosal trauma, mucosal prolapsed and comorbid infection. The third model consists of individuals with known IBD and DD who develop DD-related symptoms necessitating surgical resection or inflammation within the diverticulae secondary to IBD [1-5].

Histopathological findings

The pathologic triad of diverticular disease consists of thickening of the muscularis propria, diverticular penetration with accompanying mucosa and muscularis mucosa through the muscle and surface mucosal folds redundancy. The inflammatory spectrum of DD ranges from a mild to chronic active inflammatory reaction with accompanying vascular and mucosal changes. With the recent description of DAC, multiple reports suggested an overlap between diverticular disease and inflammatory bowel disease based on the observation of inflammatory changes in the bowel lumens of some patients with DD. This “overlap hypothesis” is based on the histological resemblance between the two diseases such as crypt inflammation, architectural abnormalities, lymphoplasmacytic invasion of the mucosa in its full thickness and mucosal expansion. Moreover, DAC can manifest the histological triad of chronic transmural inflammation, noncaseating granuloma and fissuring ulcers or fistulae which are features similar to Crohn’s disease [1-5].

Differential diagnoses

The main differential diagnosis of DAC is IBD in view of their similar clinical and histopathological features that distinguish them from most other forms of chronic colitis. Both entities can present with hematochezia, urgency, and tenesmus. Moreover, the possible association of DAC with extraintestinal manifestations including pyoderma gangrenosum, erythema nodosum, arthritis and ankylosing spondylitis confers a heavy diagnostic and therapeutic burden for caregivers in view of the overlapping features with IBD. Other diagnoses include mucosal trauma, mucosal prolapse, ischemic colitis, and infectious colitis.

DAC was previously suspected to be a manifestation of CD in view of the fact that it coincides with the 2nd peak of occurrence of CD. The results of multiple clinicopathologic studies favored the hypothesis that Crohn’s-like DAC is an idiosyncratic inflammatory manifestation of diverticular disease rather than a manifestation of CD occurring simultaneously with DD. The largest study of patients who underwent surgery for the affected colon segment showed that out of 25 patients with no evidence of CD prior or at the same time of surgery, 23 showed no evidence of CD after a median follow-up of six years. In addition, similar histological features were found in patients who developed CD within six months after surgery, patients who did not develop CD, and patients with evidence of CD in other areas of their bowels prior or at the same time as the surgery.

Few reports noted an analogy between Cohn’s-like DAC and chronic granulomatous appendicitis which shares the same histological features as CD. Very few cases of chronic granulomatous appendicitis progressed to develop overt CD. Moreover, the histological aspects of the resected appendices could not predict the occurrence of CD in these patients. Another striking similarity with Crohn’s-like DAC is that the Crohn’s-like changes in appendicitis cases were seen in the majority of patients who received antibiotic therapy and drainage for 30 to 95 days prior to resection which parallels the more frequent occurrence of Crohn’s-like DAC in patients with medically resistant diverticulitis who received prolonged courses of antibiotic therapy prior to resection. These findings support the idiosyncratic nature of DAC and the inefficacy of tissue morphology solely to diagnose CD.

In addition, some articles reported the occurrence of classical Ulcerative Colitis in patients initially diagnosed with DAC 25 months or less prior. Supporting this hypothesis is the theory of “blind pouch effect” describing a discontinuous pattern of UC in the blinded areas of the colon, notably the appendix and the cecum [1-5].

Treatment

Medications used for DAC are similar to those used in IBD. Use of 5-aminosalicylic acid has shown high success in segmental colitis. In a subset of patients, surgical intervention is warranted with studies noting low risk of recurrences after follow-up for up to seven years. Fiber supplements and antispasmodics should be given to patients with histopathological changes similar to IBD. Moreover, in patients not considered to have primary IBD, 25% of them progress to CD, 10% to UC whereas the remaining will have localized sigmoid colitis that usually responds to medical treatment [1-5].

## Conclusions

DAC is an infrequent manifestation of the inflammatory spectrum of DDs that can present with granulomatous reaction of the involved colon segment. Clinical and pathologic overlap between this entity and IBD namely Crohn’s disease poses a diagnostic and therapeutic dilemma in the management of affected individuals. In addition, rates of recurrence and long-term outcomes are not well defined ranging from a benign course to manifestations of overt IBD. More studies are needed in order to further characterize this entity.
